# Impact of Polycystic Ovary Syndrome Status on Changes in Reproductive Function During a Hypocaloric Dietary Intervention

**DOI:** 10.3390/nu18040654

**Published:** 2026-02-16

**Authors:** Jie Zhang, Joy Y. Kim, Ryan Levine, Catherine Cho, Hannah Lee, Eli Thoma, Faith E. Carter, Brittany Y. Jarrett, Bailey Smith, Heidi Vanden Brink, Marla E. Lujan

**Affiliations:** 1 Division of Nutritional Sciences, Cornell University, Ithaca, NY 14850, USA; jz2225@cornell.edu (J.Z.); jyk47@cornell.edu (J.Y.K.);; 2 Department of Nutrition, Texas A&M University, College Station, TX 77840, USA

**Keywords:** polycystic ovary syndrome, diet, caloric restriction, reproductive health

## Abstract

**Background/Objectives**: Lifestyle interventions are first-line treatment for women with polycystic ovary syndrome (PCOS) to improve metabolic health. Impacts on reproductive function are less clear. Previous research has been limited by inconsistencies in evaluation of ovulatory function and lack of comparisons between women with and without PCOS. **Methods**: The present study implemented a prospective clinical trial of 28 women (PCOS, N = 10 and Non-PCOS Control, N = 18) undergoing a 1-month baseline assessment followed by a 6-month hypocaloric dietary intervention. **Results**: Both groups reached clinically meaningful weight loss with the intervention (PCOS group: 6.5 ± 5.5%; Non-PCOS Control group: 10.0 ± 4.7%). Largest follicle diameter and growth rate of ovulatory dominant follicle, menstrual cycle length and luteal phase length did not change during the intervention in either group (all *p* > 0.05). The Non-PCOS Control group had increased mid-luteal phase progesterone levels and secretory phase maximum endometrial thickness during the intervention (all *p* < 0.05), whereas the PCOS group showed a reduction in follicular phase length (*p* < 0.05). Additionally, changes in ovulatory function and endometrial development were not associated with the rate of weight loss (all *p* > 0.05). **Conclusions**: This study demonstrates that women with PCOS are unlikely to experience changes in menstrual cyclicity and endometrial development with a short-term hypocaloric dietary intervention. The shortening of the follicular phase suggests that women with PCOS may need a longer intervention to achieve clinically meaningful improvements in ovulatory function and endometrial health.

## 1. Introduction

The prevalence of overweight and obesity among women of reproductive age has increased worldwide and can be as high as 54% in the United States [1,2]. Increased risk of menstrual irregularity and anovulatory infertility has been documented in women with obesity [3]. However, ovulatory dysfunction in women with overweight and obesity is often confounded by polycystic ovary syndrome (PCOS). Overweight and obesity are more prevalent among women with PCOS compared to the general population [4] and obesity has been noted to exacerbate reproductive dysfunction in PCOS, including likelihood of infertility and endometrial cancer [5,6]. Insulin resistance resulting from accumulation of excess visceral fat leads to compensatory hyperinsulinemia. Hyperinsulinemia contributes to hepatic suppression of sex-hormone binding globulin (SHBG) and increased free androgen bioavailability [6]. Accordingly, women with obesity alone show signs of subclinical hyperandrogenism consistent with obesity, representing a state of functional hyperandrogenism [7]. Furthermore, hypersecretion of luteinizing hormone (LH) and ovarian theca cell dysfunction are contributing factors to hyperandrogenism [8]. Collectively, interventions targeting overweight and obesity are critical for reproductive health in women of reproductive age.

Although weight loss through lifestyle intervention is the recommended first-line treatment for women with overweight/obesity and PCOS [9], it remains uncertain how lifestyle-induced weight loss impacts reproductive outcomes. Prior studies have demonstrated that clinically meaningful weight loss (5–10%) is associated with significant improvements in body composition, cardiometabolic health, and quality of life in women with and without PCOS [10,11]. However, most studies evaluating effects of weight loss in women of reproductive age have not contrasted reproductive health outcomes in women with and without ovulatory dysfunction, nor have they evaluated ovulatory function and endometrial development concurrently.

Studies that evaluated effects of lifestyle intervention on reproductive function mainly focused on assessing changes in reproductive hormone concentrations in women with reproductive dysfunction broadly defined [12]. Findings from most lifestyle intervention studies in PCOS suggests a marginal lowering of total and free testosterone levels–albeit findings are inconsistent [13,14,15]. Current practice guidelines for PCOS specify that the impact of lifestyle intervention on reproductive function is uncertain [9]. Previous studies assessing changes in ovulatory cyclicity through lifestyle intervention in PCOS are limited due to lack of age- and BMI-matched control groups, inconsistencies in ovulation measures and lack of baseline data [10]. Evaluation of a comprehensive set of reproductive health outcomes in women with and without PCOS is needed to provide new knowledge regarding expectations of improvements in reproductive function with lifestyle intervention.

Endometrial development is closely related to ovarian function and appropriate communication between the uterus and the ovaries is critical for fertility and for offsetting the risk of endometrial pathology [16,17]. Even though overweight/obesity and PCOS increase the risk for endometrial hyperplasia and endometrial cancer [18,19], there is a lack of data on endometrial development or how lifestyle intervention might differentially impact endometrial health in women with and without PCOS. Together, there is an urgent need for studies evaluating changes in ovulatory function and endometrial development in both women with and without PCOS to fully elaborate the impact of lifestyle intervention in this population.

To address this gap in knowledge, the primary objective of this study was to contrast changes in ovulatory function and endometrial development during hypocaloric dietary intervention in women with and without PCOS. Associations between changes in reproductive function and the rate of weight loss were also explored. We hypothesized that both women with and without PCOS would experience favorable changes in ovulatory function and endometrial development in response to a hypocaloric dietary intervention. We also hypothesized that women with PCOS might demonstrate a slower rate of change in ovulatory function and endometrial development compared to those without, given their underlying reproductive dysfunction.

## 2. Materials and Methods

### 2.1. Study Participants

Female participants of reproductive age (18–38 years old) with overweight or obesity (BMI ≥ 25 kg/m^2^) were recruited to a double-arm 7-month weight loss study (7MO WLS) that implemented a hypocaloric dietary intervention. Participants were recruited from the general population in Ithaca, NY and surrounding areas through circulating paper and electronic advertisements from 2018 to 2023. Briefly, participants met inclusion criteria if they had consistent, optimal ultrasound visualization of both ovaries and the uterus, and were assessed to be at the ready to lose weight stage using a validated weight loss readiness questionnaire [20]. Participants had not used hormonal contraception or fertility drugs in the two months before enrollment and were not planning to become pregnant during the full study period. Exclusion criteria included use of medications or supplements suspected to impact metabolic function in the two months prior (insulin sensitizer, weight loss medications, inositol, etc.), recent pregnancy or lactation, history of ovarian surgery or bariatric surgery, and/or presence of medical conditions that would interfere with study procedures (untreated thyroid abnormalities, hyperprolactinemia, abnormal 17-hydroxprogesterone production, diabetes mellitus, etc.).

PCOS was defined by the International Evidence-Based PCOS Guideline supported Rotterdam criteria of two or more cardinal features: (1) oligo- or anovulation; (2) clinical and/or biochemical hyperandrogenism; (3) polycystic ovaries [9]. Oligo-anovulation was determined by self-reported menstrual history of irregular menstrual cycles, which was defined as an average menstrual cycle > 35 days in the year prior to enrollment. Clinical hyperandrogenism was defined as having a modified Ferriman–Gallwey hirsutism score of ≥6 [9]. Biochemical hyperandrogenism was evaluated based on serum total testosterone ≥ 61.5 ng/dL, free androgen index (FAI) ≥ 6, free testosterone ≥ 0.82 ng/dL and/or bioavailable testosterone ≥ 19.1 ng/dL [9]. Thresholds for biochemical hyperandrogenism were derived from an independent group of healthy reproductive age women with regular menstrual cycles and represented the 95th percentiles of androgen concentrations. Polycystic ovaries were based on follicle number per ovary (FNPO) ≥ 25, and/or ovarian volume (OV) ≥ 10 mL on ultrasound [9,21]. Regular menstrual cycles were based on an average menstrual cycle of 21–35 days in the year prior to enrollment using self-reported menstrual history. Data on reproductive outcomes in a subset of participants in the Non-PCOS Control group have been previously published [22].

### 2.2. Study Design

This was a prospective lifestyle intervention study designed to evaluate the impact of androgen status on changes in menstrual cyclicity during a hypocaloric dietary intervention. We intended to recruit participants to the following groups: (1) participants with oligo-anovulation and hyperandrogenism (PCOS); (2) participants with oligo-anovulation and normal androgen levels; and (3) healthy participants with ovulatory cycles. However, we experienced difficulties in recruitment and repeated research shut-downs during the COVID-19 pandemic and were unable to reach our intended sample sizes due to depletion of funds. As a result, we modified our primary research question to contrast changes in reproductive function during a short-term hypocaloric dietary intervention in women with and without PCOS. Primary reproductive function outcomes were defined as (1) ovulatory function and (2) endometrial development. During the 7MO WLS, study participants visited the Human Metabolic Research Unit (HMRU) at Cornell University every other day in month 1 (MO1) and month 7 (MO7), and at least two times per week during month 2 (MO2) through month 6 (MO6) for a transvaginal ultrasound scan, weight measurement and venipuncture. Additionally, participants attended one early morning visit pre-intervention (MO1) and post-intervention (MO7) for the following procedures: (1) fasting blood draw; (2) conventional anthropometry; (3) hirsutism assessment; and (4) transvaginal ultrasound scan. Fasting blood sample was used to assess serum concentrations of total and free androgens, including total testosterone, SHBG and FAI. Conventional anthropometry assessed height, body weight and calculated BMI. The degree of hirsutism was evaluated using the modified Ferriman–Gallwey Scoring System [9].

### 2.3. Hypocaloric Dietary Intervention

During the pre-intervention baseline period (MO1), participants were advised to maintain their usual lifestyle habits. After baseline assessment, participants began a 6-month hypocaloric dietary intervention, which implemented a commercial meal delivery service designed to help participants follow a diet of 1250–1500 calories per day (Nutrisystem^®^ D, Nutrisystem, Inc., Fort Washington, PA, USA). This low calorie, low glycemic index meal plan included 3 entrees, 2 snacks and 1 dessert per day, supplemented with fresh fruits and vegetables to balance nutritional needs. Nutrisystem^®^ D follows the USDA Dietary Guidelines, with the meal plan consisting of 50% carbohydrate, 25% protein and 25% fat. The diet also included 25–35 g of fiber and <2300 milligrams of sodium [23]. A trained nutritionist from the research team worked with the participants to customize meal plans per individual preferences and a Registered Dietitian from Nutrisystem^®^ D facilitated compliance with the dietary intervention during the study.

### 2.4. Ultrasound Image Acquisition and Analysis

Transvaginal ultrasound scans were performed using a high-resolution GE Voluson E8 and E10 Expert System with a 6–12 MHz 3D/4D transducer (GE Healthcare, Milwaukee, WI, USA). The diameter of the largest follicle at each study visit was monitored in real time by a sonographer on the research team. When the diameter of the dominant follicle (≥10 mm) exceeded 16 mm, participants were invited to visit the HMRU for daily ultrasound scans until ovulation occurred or the dominant follicle regressed. During each examination, sweeps of both ovaries and the uterus were collected and archived as 3D volume files (.4dv) for retrospective image analysis. The 3D volume files were partitioned into individual 2D cineloops (.dcm) of the three anatomical planes and those in the sagittal and transverse planes were analyzed off-line using medical imaging software (Santesoft DICOM Editor Version 3.1.2; ©Emmanouil Kanellopoulus, Athens, Greece). Specifically, number and diameter of follicles ≥ 2 mm were measured with a programmable grid overlay to improve reliability in counts [24]. The diameter of follicles < 10 mm was calculated as the mean of its length and perpendicular width in its largest cross-sectional area in one plane and the diameter of follicles ≥ 10 mm was measured as the mean of its length and perpendicular width in its largest cross-sectional area in both the sagittal and transverse planes. Antral follicles ≥ 7 mm were individually tracked using the Identity Method, which utilized sketches of follicle location within an ovary from serial ultrasound scans to assess follicle growth trajectory [25]. Serial endometrial thickness was measured as the distance from the anterior to the posterior stratum basalis-myometrial junction within a 5–10 mm range from the tip of the fundus in the mid-sagittal plane [16]. The images were analyzed by experienced raters that showed strong inter-rater agreement in assessments of ovarian morphology and endometrial thickness based on a review of an independent set of images (ICC > 0.80). Additional endpoints assessed using scans of the ovaries included (1) FNPO and (2) OV. FNPO was the total count of all follicles per ovary. OV was calculated based on the prolate ellipsoid equation using the length, width and height of the ovary. FNPO and OV are reported as the average of the left and right ovaries.

### 2.5. Definition of Reproductive Function

Reproductive function was assessed by ovulatory function and endometrial development during the study. The primary outcome for ovulatory function was menstrual cycle length. Menstrual cycle length was defined as days between the start of two consecutive menses. Menses was defined as bleeding of ≥2 days in a 3-day bleeding interval preceded by ≥2 days free of bleeding. Bleeding of >7 days was not defined as menses, which did not include abnormal uterine bleeding. Secondary outcomes included the growth dynamics of ovulatory dominant follicles (largest follicle size and growth rate). Length of the follicular phase (follicular phase length) and luteal phase (luteal phase length) were based on documentation of menses and ovulation during the study interval. Follicular phase length was defined as the duration from the first day of menses to the day of ovulation. Ovulation was defined as the disappearance of a dominant follicle followed by the visualization of a corpus luteum (CL) on ultrasonography and was confirmed post hoc by a rise in serum progesterone concentrations of ≥1.5 ng/mL. Luteal phase length was defined by the time after ovulation to the day prior to the onset of the subsequent menses. Progesterone levels were assessed at the mid-luteal phase to evaluate luteal function. The primary outcome for evaluating endometrial development was endometrial thickness (minimum and maximum thickness), which was measured in the proliferative and secretory phases of the uterine cycle. These correspond to the follicular and luteal phases of the ovarian cycle, respectively.

### 2.6. Biochemical Analysis

Blood samples were processed and serum specimens were stored at −80 °C. Total testosterone was measured at a clinical chemistry laboratory that participates in the Centers for Disease Control and Prevention (CDC) Hormone Standardization (HoST) Program using liquid chromatography tandem mass spectrometry (Brigham Research Assay Core Laboratory, Boston, MA, USA). SHBG, LH, and serum follicle-stimulating hormone (FSH) were measured in-house by chemiluminescence immunoassays (Immulite 2000; Siemens Medical Solutions Diagnostics, Deerfield, IL, USA). FAI was calculated as: ([total testosterone] ÷ [SHBG]) × 100. Anti-Mullerian hormone (AMH) was measured at a commercial laboratory using enzyme-linked immunosorbent assay (Ansh Labs, Webster, TX, USA). Inter-assay variation was <10% across all in-house assays.

### 2.7. Statistical Analysis

All statistical analyses were performed using RStudio Version 2024.12.1+563 (RStudio: Integrated Development for R. RStudio, PBC, Boston, MA, USA). Statistical significance was defined by *p* < 0.050. Normality was assessed using histograms and residual plots, and data were log-transformed if not normally distributed. Cross-sectional data were compared between groups using Wilcoxon rank-sum test. Fisher’s exact tests were used to compare cross-sectional categorical data between groups. Holm’s method was used to control the family-wise error rate across multiple pairwise comparisons. Differences in androgen status markers and reproductive hormone (i.e., hirsutism score, total testosterone, FAI, SHBG, LH, FSH, LH:FSH ratio and AMH) pre- and post-intervention were compared using pairwise Wilcoxon rank-sum test. Linear mixed models were used to evaluate longitudinal changes in reproductive function markers between groups. Model assumptions were assessed using residual diagnostics. Outcomes with skewed residual distributions were log-transformed prior to analysis, whereas approximately normally distributed outcomes were analyzed on their original scale. Outcomes that were log-transformed included menstrual cycle length, dominant follicle growth rate, and follicular phase length. Models included a random intercept for participant and a random slope for intervention week. Fixed effects included intervention week, PCOS status and their interaction. Post hoc trend analyses were conducted to estimate and compare group-specific slopes. Overall group differences across the 24-week intervention period were assessed using estimated marginal means. To quantify the magnitude of the differences, standardized effect sizes (Cohen’s d) were calculated by dividing the estimated marginal means differences by the model-based residual standard deviation. 95% confidence intervals (CIs) and *p*-values were reported for all comparisons. Statistical significance was set at α = 0.05. Pearson’s correlation coefficient was calculated to assess correlation between changes in reproductive function markers and the rate of weight loss during the intervention.

## 3. Results

### 3.1. Participant Characteristics

**Figure 1:**
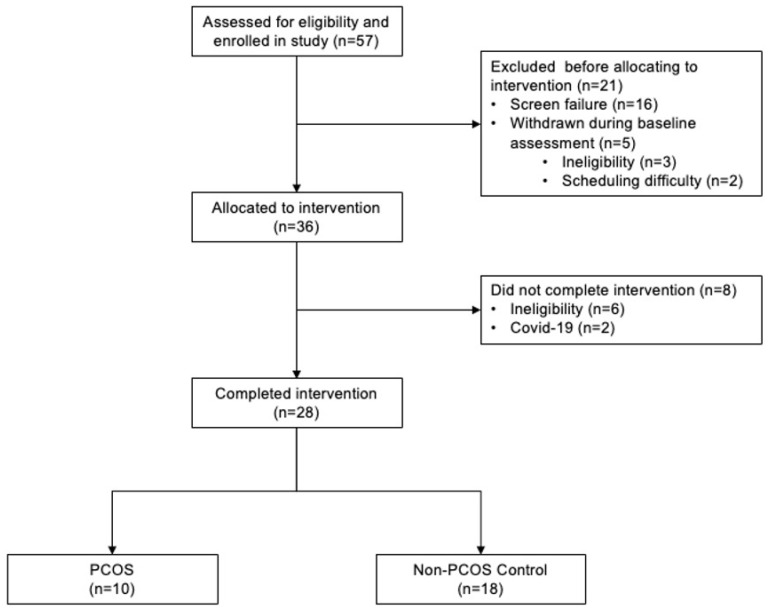
Flow of the study participants through a clinical trial evaluating the impact of polycystic ovary syndrome (PCOS) status on changes in reproductive function during a hypocaloric dietary intervention. Twenty-eight participants completed the full study. Of the 28 participants, 10 participants met the criteria for PCOS and the remaining 18 participants with regular menstrual cycles met criteria for controls.

A schematic summarizing participant flow during the clinical trial is presented in Figure 1. Fifty-seven participants enrolled in the clinical trial. Sixteen participants were excluded due to screen failure (i.e., poor visualization of the ovaries and the uterus, poor vein palpability) and five were withdrawn from the trial during the 1-month baseline assessment due to ineligibility (i.e., consistently poor visualization of the ovaries, high fasting glucose level) and scheduling difficulty. Of the thirty-six participants who completed the pre-intervention baseline month and were allocated to the dietary intervention, eight participants were withdrawn during the intervention due to ineligibility (i.e., consistently poor visualization of the ovaries, scar tissue build-up of veins, unsuccessful weight loss, difficulty adopting to Nutrisystem^®^ D and starting new medication suspected to interfere with reproductive function) and COVID-19 pandemic-related research shutdowns. Ultimately, 28 participants completed both the pre-intervention baseline assessment and the full intervention (22% attrition). Baseline characteristics were contrasted between participants that completed the intervention (Study Completion; N = 28) and those that did not complete the intervention (Study Withdrawal; N = 8) in Supplemental Table S1. Participants in the Study Withdrawal group had larger OV at baseline (*p* < 0.05). All other characteristics were comparable between the two groups (all *p* > 0.05). Of the 28 participants who completed the entirety of the study, 10 participants met the criteria for PCOS [9] and the remaining 18 participants with regular menstrual cycles met criteria for controls.

Baseline characteristics of the study participants (N = 28) are compared between groups in Table 1. Groups did not differ in terms of age, race, ethnicity, body weight and BMI (all *p* > 0.05). Menstrual cycle length, hirsutism score, total testosterone and FAI were higher in the PCOS group at baseline compared to the Non-PCOS Control group by design (all *p* < 0.05). FNPO and OV were similar between women with PCOS and the Non-PCOS Control group (all *p* > 0.05).

### 3.2. Androgen Status Markers and Reproductive Hormones

Comparisons of androgen status markers and reproductive hormones pre- and post-intervention and between groups are reported in Table 2. In addition to baseline markers described in Table 1, the PCOS group had significantly lower SHBG compared to the Non-PCOS Control group (*p* < 0.05). At baseline, groups did not differ in FSH (*p* > 0.05). LH, LH:FSH ratio and AMH were higher in the PCOS group compared to the Non-PCOS Control group at baseline (all *p* < 0.05). Post-intervention, the PCOS and the Non-PCOS Control groups had similar total testosterone, LH, FSH and LH:FSH ratio (all *p* > 0.05). Hirsutism score, FAI and AMH remained higher and SHBG remained lower in the PCOS group compared to the Non-PCOS Control group post-intervention (all *p* < 0.05). Androgen status markers and reproductive hormones pre- and post-intervention did not differ in either group (all *p* > 0.05).

### 3.3. Ovulatory Function

**Figure 2:**
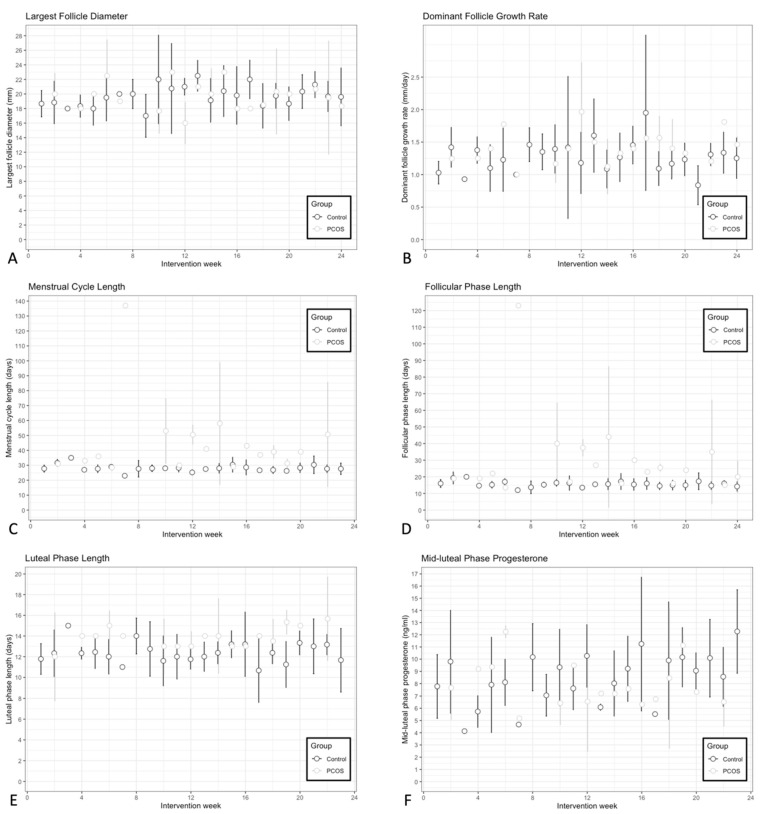
Longitudinal assessments of largest follicle diameter (**A**), dominant follicle growth rate (**B**), menstrual cycle length (**C**), follicular phase length (**D**), luteal phase length (**E**) and mid-luteal progesterone (**F**) during the intervention in the PCOS (light gray O) and the Non-PCOS Control group (black O). Mixed models showed a group effect for menstrual cycle length (log-transformed) and follicular phase length (log-transformed), and a time effect for mid-luteal phase progesterone.

Longitudinal assessments of ovulatory function markers during the intervention are plotted in Figure 2. Four to seven ovulations were documented per participant in the Non-PCOS Control group during the intervention. Nine out of the ten participants with PCOS had at least two ovulations during the intervention. One participant in the PCOS group did not experience ovulation or menses during the intervention and therefore was not included in the assessment of reproductive function markers (ovulatory function and endometrial development). At baseline, menstrual cycle length was longer in the PCOS group compared to the control group, as expected (estimate: 0.697, *p* < 0.01). It did not change significantly during the intervention for either group (Control slope: −0.002, 95% CI [−0.014, 0.011]; PCOS slope: −0.017, 95% CI [−0.036, 0.003]). However, there was an overall difference in cycle length between groups, with PCOS participants maintaining significantly longer cycles throughout the study (estimate: −0.519, 95% CI [−0.729, −0.31], Cohen’s d = −2.16, *p* < 0.0001) (Figure 2C). At baseline, the PCOS group had a longer follicular phase (estimate: 0.879, *p* < 0.01) and was the only group to experience a significant shortening of folllicular phase length (Control slope: −0.004, 95% CI [−0.015, 0.006]; PCOS slope: −0.023, 95% CI [−0.041, −0.006]). Throughout the intervention, a significant difference in follicular phase length remained with the PCOS group maintaing a longer follicular phase (estimate: −0.639, 95% CI [−0.914, −0.364], Cohen’s d = −2.46, *p* < 0.0001) (Figure 2B).

Largest follicle diameter (Control slope: 0.0632, 95% CI [−0.009, 0.136]; PCOS slope: 0.014, 95% CI [−0.125, 0.153]; (Figure 2A), dominant follicle growth rate (Control slope: 0.002, 95% CI [−0.007, 0.012]; PCOS slope: 0.005, 95% CI [−0.012, 0.022]; Figure 2B) and luteal phase length (Control slope: 0.018, 95% CI [−0.044, 0.079]; PCOS slope: 0.098, 95% CI [−0.018, 0.213] Figure 2E) did not change during the intervention for either group. However, although there was no difference in the length of the luteal phase at baseline between groups, there was a difference during the intervention, with the PCOS group having a longer luteal phase (estimate: −1.27, 95% CI [−2.43, −0.100], Cohen’s d = −0.341, *p* < 0.05). There were no differences in largest follicle diameter (estimate: 0.081, 95% CI [−1.73, 1.89], *p* > 0.05) and dominant follicle growth rate (estimate: −0.133, 95% CI [−2.82, 0.0152], *p* > 0.05) between groups during the intervention. Additionaly, mid-luteal progesterone levels increased during the intervention for the Non-PCOS Control group, but not the PCOS group (Control slope: 0.091, 95% CI [0.009, 0.173]; PCOS slope: −0.020, 95% CI [−0.187, 0.148]; Figure 2F). However, there was no difference between the groups (estimate: 1.04, 95% CI [−0.861, 2.95], *p* > 0.05).

**Figure 3:**
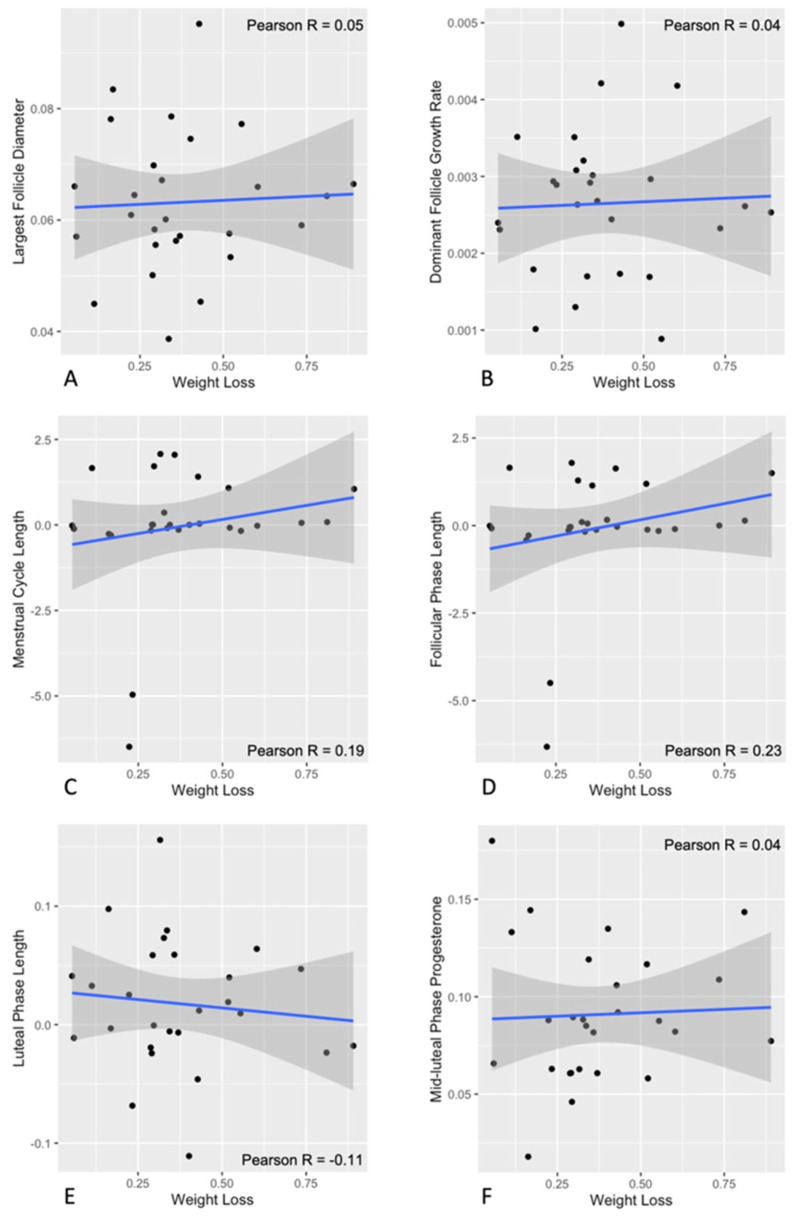
Scatter plots with Pearson’s correlation coefficient between changes in ovulatory function markers and the rate of weight loss. Correlation between changes in largest follicle diameter (**A**), dominant follicle growth rate (**B**), menstrual cycle length (**C**), follicular phase length (**D**), luteal phase length (**E**), mid-luteal phase progesterone (**F**) and the rate of weight loss are presented. Pearson’s correlation coefficients are shown (Pearson R). No significant (*p* < 0.05) associations were noted.

All participants reached clinically meaningful weight loss post-intervention (6.5 ± 5.5% in the PCOS group and 10.0 ± 4.7% in the Non-PCOS Control group). Scatter plots of changes in ovulatory function markers and the rate of weight loss during the intervention are shown in Figure 3. Changes in menstrual cycle length (Figure 3C), largest follicle diameter (Figure 3A), dominant follicle growth rate (Figure 3B), follicular phase length (Figure 3D), luteal phase length, (Figure 3E) and mid-luteal progesterone levels (Figure 3F) were not associated with the rate of weight loss (all *p* > 0.05).

### 3.4. Endometrial Development

**Figure 4:**
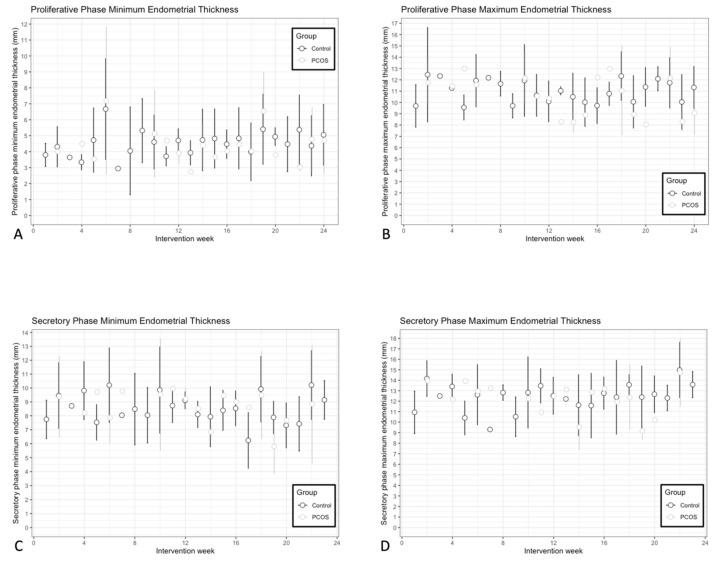
Longitudinal assessments of proliferative phase minimum endometrial thickness (**A**), proliferative phase maximum endometrial thickness (**B**), secretory phase minimum endometrial thickness (**C**), and secretory phase maximum endometrial thickness (**D**) during the intervention in the PCOS (light gray O) and the Non-PCOS Control group (black O). Mixed models showed a time effect and an interaction group*time effect for secretory phase maximum endometrial thickness.

**Figure 5:**
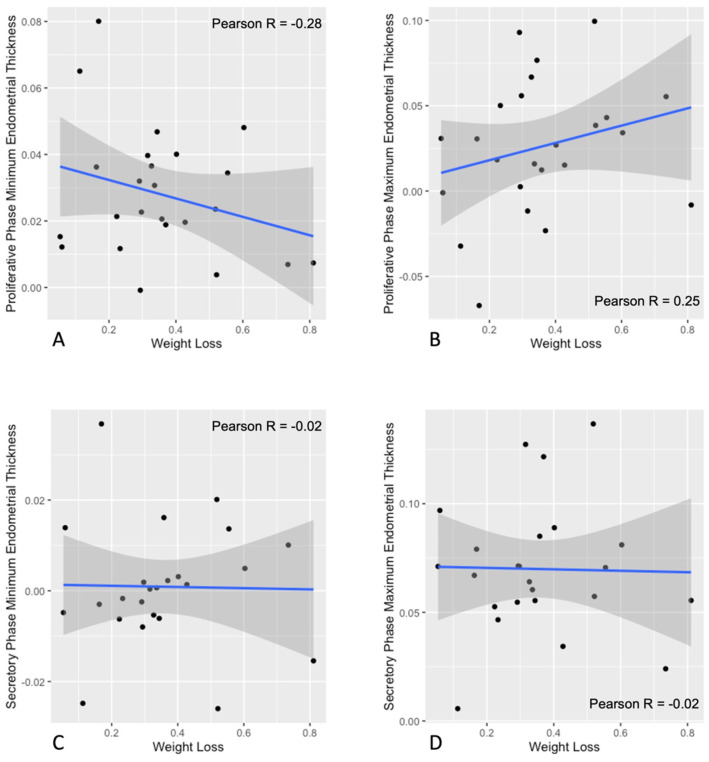
Scatter plots with Pearson’s correlation coefficient between changes in ovulatory function markers and the rate of weight loss. Correlation between changes in proliferative phase minimum endometrial thickness (**A**), proliferative phase maximum endometrial thickness (**B**), secretory phase minimum endometrial thickness (**C**), secretory phase maximum endometrial thickness (**D**) and the rate of weight loss are presented. Pearson’s correlation coefficients are shown (Pearson R). No significant (*p <* 0.05) associations were noted.

Serial assessments of endometrial development markers during the intervention are plotted in Figure 4. Proliferative phase minimum endometrial thickness (Control slope: 0.028, 95% CI [−0.012, 0.068]; PCOS slope: −0.049, 95% CI [−0.124, 0.027]); (Figure 4A), proliferative phase maximum endometrial thickness (Control slope: 0.026, 95% CI [−0.023, 0.075]; PCOS slope: −0.073, 95% CI [−0.163, 0.016]); (Figure 4B), and secretory phase minimum endometrial thickness (Control slope: 0.001, 95% CI [−0.043, 0.044]; PCOS slope: −0.048, 95% CI [−0.135, 0.040]); (Figure 4C) did not change during the intervention for either group. None of these endometrial outcomes differed between groups during the intervention as well (proliferative phase minimum endometrial thickness: estimate: 0.172, 95% CI [−1.020, 1.370]; proliferative phase maximum endometrial thickness: estimate: 0.078, 95% CI [−1.530, 1.690]; secretory phase minimum endometrial thickness: estimate: −0.11, 95% CI [−1.780, 1.560]; all *p* > 0.05). In contrast, secretory phase maximum endometrial thickness increased during the intervention for the Non-PCOS Control group, but not the PCOS group (Control slope: 0.070, 95% CI [0.018, 0.122]; PCOS slope: −0.068, 9% CI [−0.165, 0.029]) (Figure 4D). The measure did not differ between groups (estimate: −0.11, 95% CI [−1.780, 1.560], *p* > 0.05). Scatter plots of changes in endometrial development markers and the rate of weight loss during the intervention are shown in Figure 5. There was no association between changes in endometrial thickness during the proliferative phase or the secretory phase and the rate of weight loss (all *p* > 0.05) (Figure 5A–D).

## 4. Discussion

The present study provides a novel comparison of changes in reproductive function in women with and without PCOS during a short-term hypocaloric dietary intervention. We showed that women with PCOS experienced a shortening of the follicular phase, albeit this did not translate into meaningful changes in menstrual cycle length. Additionally, only the Control group had had increased mid-luteal phase progesterone levels and secretory phase maximum endometrial thickness during the intervention. By contrast, menstrual cycle length, growth dynamics of ovulatory dominant follicles and proliferative phase endometrial development were not altered during short-term hypocaloric dietary intervention. Changes in reproductive function markers were not associated with the rate of weight loss, suggesting that these outcomes were independent of magnitude of weight loss. Collectively, this new knowledge suggests that women with PCOS are unlikely to experience changes in ovulatory function and endometrial development in response to short-term hypocaloric dietary intervention compared to their non-PCOS counterparts.

Overweight and obesity adversely impact reproductive function [26,27]. Decreased luteal phase progesterone [28,29], shortened luteal phase length [30] and disordered antral follicle development [29] have been reported in women with regular menstrual cycles, and may underlie the reduced fecundity characteristic of women with overweight and obesity. To date, most studies assessing impacts of lifestyle intervention on reproductive function in women of reproductive age have mainly included heterogeneous populations with ovulatory dysfunction or infertility [12]. Data in women with obesity and regular menstrual cycles are scarce and show inconsistent findings regarding changes in antral follicle development [22] and reproductive hormone concentrations, including improvements in luteal phase progesterone concentrations post-intervention [22,31,32,33]. In the current study, women with PCOS experienced a shortening of the follicular phase. This supports previous research that has shown that antral follicle development appears more cyclic with decreased frequency of recruitment following short-term hypocaloric intervention [34]. Despite a shortening of the follicular phase, no other improvements in ovulatory function were observed in the PCOS group. By contrast, mid-luteal phase progesterone levels increased during the intervention for the Non-PCOS Controls, irrespective of the rate of weight loss. This suggests improvement in luteal function with hypocaloric dietary intervention over time. This increase in progesterone levels may have been driven by a transition to follicle selection at a more normal size with weight loss as previously reported in this control group [22]. Size at selection is associated with improvement in luteinization after ovulation as judged by increased progesterone production [22,35].

The International Evidence-Based Guideline for the Assessment and Management of PCOS suggests that lifestyle interventions be recommended to all women with PCOS for improvements in metabolic health [9]. However, there is not sufficient evidence to support improvements in hormonal and reproductive outcomes with dietary intervention [9]. To date, few studies assessing changes in reproductive outcomes with lifestyle intervention in PCOS have included ovulation or menstrual cyclicity as primary outcomes [36,37,38]. It has been difficult to harmonize data for the purposes of developing evidence-based recommendations on the impact of PCOS status on reproductive outcomes during lifestyle interventions. This has largely been due to inconsistencies in study designs and methods to evaluate baseline cycle status and/or ovulation, and lack of age- and BMI-matched control groups [9,10]. Findings from the present study align with current PCOS practice guidelines in that a short-term hypocaloric dietary intervention was not sufficient for improvements across a majority of ovulatory function markers (menstrual cycle length, growth dynamics of ovulatory dominant follicles). However, sustained healthy eating could have reproductive health benefits in PCOS as evidenced by shortening of the follicular phase during the short-term hypocaloric intervention. More detailed analyses of the antral follicle dynamics would more strongly support this conclusion.

In addition to ovulatory function, we assessed endometrial development to comprehensively evaluate changes in reproductive function during the intervention. We had hypothesized that proliferative phase endometrial thickness would be lower with weight loss during the short-term hypocaloric dietary intervention, aligning with a transition to an increased frequency of menses in women with PCOS. We noticed that proliferative phase minimum and maximum endometrial thickness did not change during the intervention in either women with or without PCOS. No change in endometrial development during the proliferative phase aligns with the lack of an impact of the hypocaloric dietary intervention on reproductive hormone concentrations or menstrual cycle length. By contrast, we showed that secretory phase maximum endometrial thickness increased during the intervention for women without PCOS. Few data are available on endometrial thickness across the uterine cycle in women of reproductive age during natural cycles and the cutoffs for normal endometrial thickness remain controversial [39]. Alterations in the expressions of steroid hormone receptors of the endometrium have been reported in women with PCOS [40,41,42]. Studies assessing changes in gene and protein expressions of progesterone receptors (progesterone receptor A and progesterone receptor B) in women with obesity and PCOS showed improvements, but not full restoration, in endometrial function with lifestyle intervention [42]. We interpret our findings to mean that changes in progesterone induced by short-term hypocaloric dietary intervention had functional consequences for endometrial development for the Non-PCOS Control group, albeit the clinical relevance of these modest increases is uncertain.

Our intensive study protocol enabled evaluation of differences in longitudinal changes in ovulatory function and endometrial development by PCOS status during a short-term hypocaloric dietary intervention. Despite a longer menstrual cycle length and a longer follicular phase length, women with PCOS had similar luteal phase length compared to the Non-PCOS Control group when ovulation occurred during the intervention. In women with PCOS, the length of the menstrual cycle did not change during the intervention, aligning with our conclusion that a 6-month hypocaloric dietary intervention was not sufficient to consistently improve menstrual cyclicity. We continuously monitored the largest follicle diameter during the intervention and did not note any change in maximum diameter achieved of ovulatory dominant follicles. This is consistent with our previous findings characterizing antral follicle dynamics with short-term hypocaloric dietary intervention in which we demonstrated that improvements in folliculogenesis were limited to the early stages of antral follicle development, namely recruitment [22,43].

Short-term hypocaloric dietary intervention was not associated with changes in androgen status markers or reproductive hormone levels in women with PCOS, coinciding with the lack of change in menstrual cycle length and follicle growth dynamic during the intervention. Previous studies evaluating changes in reproductive function with lifestyle interventions have reported lowered total or free testosterone levels post-intervention in women with PCOS [13,14,15]. Most participants in the aforementioned studies had greater degrees of hyperandrogenism at baseline. By contrast, participants with PCOS in our study had normal total testosterone levels and modest increases in FAI and clinical hirsutism scores at baseline. As such, the participants included in this study had a relatively mild PCOS presentation. Thus, only moderate improvements in androgen status marker were possible with the intervention.

This study was strengthened by the intensity of study visits. Serial ultrasound scans of both ovaries and the uterus enabled us to evaluate longitudinal changes in a variety of reproductive function markers during the entire intervention. In using a mixed model, we compared changes in reproductive function in women with and without PCOS and were able to detect similar changes in ovulatory function in response to the dietary intervention. Additionally, serial weight assessment enabled us to evaluate the association between changes in reproductive function markers and the rate of weight loss, allowing us to test whether changes in reproductive function were the results of weight loss in either group. A limitation of the study includes a non-randomized design and a small sample size. Thus, pre- versus post-intervention comparisons were likely underpowered. The dietary intervention was short-term in nature [44], and whether PCOS status affects changes in reproductive function during sustained dietary intervention or weight loss is unknown. We acknowledge that we did not assess gene or protein expression of markers of endometrial function, and therefore, the individual contribution of androgen, estrogen and progesterone receptors to changes in endometrial development could not be evaluated.

## 5. Conclusions

In summary, short-term hypocaloric dietary intervention is unlikely to improve menstrual cyclity and endometrial development in women with PCOS, which has implications for lifestyle counseling. Changes in reproductive function may not depend on the rate of weight loss, suggesting that benefits in reproductive function could be observed with lifestyle intervention over time. Future studies with a longer intervention are needed to fully understand the impact of PCOS status on sustained changes in reproductive function in the long term.

## Figures and Tables

**Table 1:** Baseline characteristics of the study participants.

	All Participants	PCOS	Non-PCOS Controls	*p*-Value
Participant (N)	28	10	18	
Age (years)	30 ± 4	28 ± 5	31 ± 3	NS
**Race**				
White	22, 78.6%	6, 60.0%	16, 88.9%	
Black	2, 7.1%	1, 10.0%	1, 5.6%	
Asian	2, 7.1%	2, 20.0%	0, 0.0%	
Other	2, 7.1%	1, 10.0%	1, 5.6%	
**Ethnicity**				
Hispanic or Latino	3, 10.7%	2, 20.0%	1, 5.6%	
Not Hispanic or Latino	25, 89.3%	8, 80.0%	17, 94.4%	
Other	1, 2.2%	0, 0.0%	0, 0.0%	
**Anthropometric Markers**				
Body Weight (kg)	99.1 ± 17.8	95.8 ± 13.7	100.9 ± 19.8	NS
BMI (kg/m^2^)	36.7 ± 6.0	35.6 ± 5.2	37.3 ± 6.5	NS
**PCOS Status Markers**				
MCL (days)	37 ± 14	51 ± 15	30 ± 2	***
Hirsutism Score	3 ± 3	5 ± 4	2 ± 2	*
Total Testosterone (ng/dL)	22.3 ± 16.5	33.2 ± 22.6	16.3 ± 7.6	*
FAI	3 ± 4	6 ± 5	1 ± 1	***
FNPO	28 ± 15	36 ± 18	24 ± 11	NS
OV (cm^3^)	7.43 ± 3.35	8.97 ± 4.22	6.58 ± 2.49	NS

Data presented as mean ± standard deviation or N with proportion (%). Within rows, * denote significant differences between groups, * *p* < 0.05, *** *p* < 0.001. Continuous data: *p*-value from Wilcoxon rank-sum test, *p*-value adjusted using Holm method. Categorical data: *p*-value from Fisher’s exact test. Abbreviations: BMI, body mass index; MCL, menstrual cycle length; FAI, free androgen index; FNPO, follicle number per ovary; OV, ovarian volume.

**Table 2:** Comparisons of androgen status markers and reproductive hormones pre- and post-intervention.

	Pre-Intervention			Post-Intervention		
	All Participants (N = 28)	PCOS (N = 10)	Non-PCOS Controls (N = 18)	All Participants (N = 28)	PCOS (N = 10)	Non-PCOS Controls (N = 18)
**Androgen Status Markers**						
Hirsutism Score	3 ± 3	5 ± 4 ^a^	2 ± 2 ^b^	3 ± 3	5 ± 4 ^c^	2 ± 2 ^d^
Total Testosterone (ng/dL)	22.3 ± 16.5	33.2 ± 22.6 ^a^	16.3 ± 7.6 ^b^	22.3 ± 8.8	25.2 ± 8.7	20.7 ± 8.7
FAI	3 ± 4	6 ± 5 ^a^	1 ± 1 ^b^	3 ± 3	4 ± 4 ^c^	2 ± 1 ^d^
SHBG (nmol/L)	38.1 ± 22.7	28.5 ± 14.7 ^a^	43.5 ± 24.9 ^b^	45.4 ± 25.0	32.0 ± 12.2 ^c^	52.8 ± 27.4 ^d^
**Reproductive Hormones**						
LH (mIU/mL)	5.52 ± 2.86	7.56 ± 3.16 ^a^	4.38 ± 1.97 ^b^	4.85 ± 3.15	5.79 ± 4.84	4.32 ± 1.60
FSH (mIU/mL)	5.90 ± 2.24	5.41 ± 1.52	6.17 ± 2.55	6.02 ± 1.66	5.45 ± 1.43	6.34 ± 1.73
LH:FSH	1.0 ± 0.6	1.5 ± 0.6 ^a^	0.8 ± 0.5 ^b^	0.8 ± 0.5	1.0 ± 0.8	0.7 ± 0.3
AMH (ng/mL)	6.54 ± 5.24	10.39 ± 6.36 ^a^	4.41 ± 2.94 ^b^	6.53 ± 4.80	9.40 ± 5.71 ^c^	4.93 ± 3.45 ^d^

Data presented as mean ± standard deviation. Differences between groups denoted by different letters pre-intervention (a, b) and post-intervention (c, d), *p*-value adjusted using the Holm method (*p* < 0.05). Abbreviations: FAI, free androgen index; SHBG, sex hormone binding globulin; LH, luteinizing hormone; FSH, follicle stimulating hormone; AMH, anti-mullerian hormone.

## Data Availability

The data are not publicly available due to privacy restrictions. The data and material underlying this article will be shared on reasonable request to the corresponding author.

## Supplementary Materials

The following supporting information can be downloaded at: https://www.mdpi.com/article/10.3390/nu18040654/s1, Table S1: Comparison of baseline characteristics of participants who completed the intervention (Study Completion) versus participants who did not complete the intervention (Study Withdrawal).

## Abbreviations

The following abbreviations are used in this manuscript:

PCOS	Polycystic ovary syndrome
SHBG	sex-hormone binding globulin
LH	Luteinizing hormone
FSH	Follicle stimulating hormone
7MO WLS	7-month weight loss study
FAI	Free androgen index
FNPO	Follicle number per ovary
OV	Ovarian volume
HMRU	Human Metabolic Research Unit
MO1	Month 1
MO7	Month 7
MO2	Month 2
MO6	Month 6
BMI	Body mass index
GnRH	Gonadotropin-releasing hormone
AMH	Anti-müllerian hormone
