# Supplementary material for: Impact of Polycystic Ovary Syndrome Status on Changes in Reproductive Function During a Hypocaloric Dietary Intervention
Source: Nutrients. 2026 Feb 16;18(4):654. doi: 10.3390/nu18040654 (PMC12943252; doi:10.3390/nu18040654)
Supplement: Supplementary file 1 [file nutrients-18-00654-s001.zip › nutrients-4112162-supplementary.pdf]

Supplemental Table S1. Comparison of baseline characteristics of participants who completed the intervention (Study Completion) versus participants who did not complete the intervention (Study Withdrawal).

	Study Completion	Study Withdrawal	p-value
Participant (N)	28	8	
Age (years)	30 ± 4	28 ± 5	NS
**Race**			
White	22, 78.6%	8, 100.0%	
Black	2, 7.1%	0, 0.0%	
Asian	2, 7.1%	0, 0.0%	
Other	2, 7.1%	0, 0.0%	
**Ethnicity**			
Hispanic or Latino	3, 10.7%	0, 0.0%	
Not Hispanic or Latino	25, 89.3%	8, 100.0%	
Other	0, 0.0%	0, 0.0%	
**Anthropometric Markers**			
Body Weight (kg)	99.1 ± 17.8	98.8 ± 13.8	NS
BMI (kg/m^2)	36.7 ± 6.0	35.8 ± 2.6	NS
**PCOS Status Markers**			
MCL (days)	37 ± 14	36 ± 11	NS
Hirsutism Score	3 ± 3	6 ± 6	NS
Total Testosterone (ng/dL)†	22.3 ± 16.5	22.8 ± 11.0	NS
FAI†	3 ± 4	2 ± 2	NS
FNPO	28 ± 15	32 ± 17	NS
OV (cm^3)	7.43 ± 3.35	10.13 ± 3.25	*

Data presented as mean ± standard deviation[1] or N with proportion (%)[2].
Within rows, * denote significant differences between groups, *P<0.05, **P<0.01, ***P<0.001.
1 p-value from Wilcoxon rank-sum test, p-value adjusted using Holm method.
2 p-value from Fisher's exact test.
Abbreviations: BMI, body mass index; MCL, menstrual cycle length; FAI, free androgen index; FNPO, follicle number per ovary; OV, ovarian volume.
† Total testosterone and FAI assessed in a total of 35 participants (Completion N=28; Withdrawal N=7) due to missing data.
